# Structural and Catalytic Characterization of a Fungal Baeyer-Villiger Monooxygenase

**DOI:** 10.1371/journal.pone.0160186

**Published:** 2016-07-29

**Authors:** Felix Martin Ferroni, Carmien Tolmie, Martha Sophia Smit, Diederik Johannes Opperman

**Affiliations:** Department of Biotechnology, University of the Free State, Bloemfontein, South Africa; Universitetet i Bergen, NORWAY

## Abstract

Baeyer-Villiger monooxygenases (BVMOs) are biocatalysts that convert ketones to esters. Due to their high regio-, stereo- and enantioselectivity and ability to catalyse these reactions under mild conditions, they have gained interest as alternatives to chemical Baeyer-Villiger catalysts. Despite their widespread occurrence within the fungal kingdom, most of the currently characterized BVMOs are from bacterial origin. Here we report the catalytic and structural characterization of BVMO_AFL838_ from *Aspergillus flavus*. BVMO_AFL838_ converts linear and aryl ketones with high regioselectivity. Steady-state kinetics revealed BVMO_AFL838_ to show significant substrate inhibition with phenylacetone, which was more pronounced at low pH, enzyme and buffer concentrations. *Para* substitutions on the phenyl group significantly improved substrate affinity and increased turnover frequencies. Steady-state kinetics revealed BVMO_AFL838_ to preferentially oxidize aliphatic ketones and aryl ketones when the phenyl group are separated by at least two carbons from the carbonyl group. The X-ray crystal structure, the first of a fungal BVMO, was determined at 1.9 Å and revealed the typical overall fold seen in type I bacterial BVMOs. The active site Arg and Asp are conserved, with the Arg found in the “in” position. Similar to phenylacetone monooxygenase (PAMO), a two residue insert relative to cyclohexanone monooxygenase (CHMO) forms a bulge within the active site. Approximately half of the “variable” loop is folded into a short α-helix and covers part of the active site entry channel in the non-NADPH bound structure. This study adds to the current efforts to rationalize the substrate scope of BVMOs through comparative catalytic and structural investigation of different BVMOs.

## Introduction

Baeyer-Villiger monooxygenases (BVMOs) are flavin-dependent enzymes that catalyze the conversion of ketones to esters using NAD(P)H and molecular oxygen [1–4]. In addition to this typical reaction, they can also catalyze heteroatom oxidation, including sulfoxidation and N-oxidation, as well as epoxidation reactions. The substrate scope of the collective BVMO family of enzymes has grown to include a variety of substrates ranging from acetone to larger ketones such as steroids. The mild reaction conditions and often high regio-, stereo- and enantioselectivity have made them very attractive as an alternative to chemical Baeyer-Villliger catalysts. Indeed, many directed evolution studies have been performed to increase or alter the substrate scope as well as improve the selectivity and specificity of these enzymes [5,6].

Although the available cloned BVMOs have grown significantly over the past few years, it is only recently that BVMOs from fungal sources have been explored [7,8] despite their wide-spread presence in the fungal-kingdom as revealed through whole-genome sequencing [9]. To date however, the three-dimensional crystal structures of only four distinct bacterial type I Baeyer-Villiger monooxygenases have been determined: phenylacetone monooxygenase (PAMO) from *Thermobifida fusca* [10], cyclohexanone monooxygenase (CHMO) from *Rhodococcus* sp. strain HI-31 [11], steroid monooxygenase (STMO) from *Rhodococcus rhodochrous* [12] and 2-oxo-Δ^3^–4,5,5-trimethylcyclopentenylacetyl-coenzyme A monooxygenase (OTEMO) from *Pseudomonas putida* ATCC 17453 [13]. Through extensive structural investigations of these enzymes with bound co-factors, inhibitors, substrates and products, the reaction mechanism of BVMOs has been explained [14–16]. Catalysis of BVMOs involves extensive backbone conformational changes and cofactor movement. In short, NADPH is bound to the BVMO in the “open” conformation, where after the non-covalently bound FAD is reduced and subsequently reacts with molecular oxygen to form the reactive peroxyflavin species. Following substrate entry, the BVMO undergoes a domain rotation and movement of the NADP^+^ to stabilize the peroxyflavin. This is accompanied/mediated by the structuring of a disordered surface loop. The BVMO, now in a “closed/tight” conformation, reorganizes to the “rotated” conformation through the rotation of the NADP^+^ to allow the substrate to move into the catalytic position. Nucleophilic attack with formation of the Criegee intermediate occurs in this “rotated” conformation. Following the production of the lactone product, the BVMO returns to a “closed/tight”-like NADP^+^ conformation followed by release of the product in the “loose” conformation.

Despite these informative studies, the basis of substrate acceptance and specificity, especially of larger substrates, is still not completely understood. PAMO has a rather limited substrate scope of mostly phenyl substituted linear ketones [17] while STMO can only convert both progesterone and phenylacetone [12,18]. In contrast CHMO has an extremely wide substrate scope [3]. We have recently reported on four closely related BVMOs from *Aspergillus flavus* with distinct substrate profiles [7]. Amongst the four BVMOs described, BVMO_AFL838_ showed the best conversion of alkanones with chain lengths of C8-C12, but was unable to convert most of the cyclic ketones tested. Here we describe the catalytic and structural characterization of BVMO_AFL838_. This structure represents the first fungal BVMO solved and contributes to the efforts to rationalize the substrate specificity of BVMOs.

## Materials and Methods

### Strains and Vectors

BVMO_AFL838_ was heterologously expressed from the pET-22b(+) vector (Novagen) in *E*. *coli* BL21Gold(DE3) (Stratagene). The previously constructed plasmid [7] served as a template to construct a C-terminally histidine (CTH) tagged variant of BVMO_AFL838_ by deleting the stop codon and plasmid backbone between the gene and the plasmid’s six histidine’s codons. This variant was prepared as previously described [19] using the primers BVMO_Histag_F (5’ CAC CAC CAC CAC CAC CAC TGA GAT C 3’) and BVMO_Histag_R (5’ TGC TTT CGC AAA ACC AAA GAA ATC CTC 3’). *Bacillus megaterium* glucose dehydrogenase (*Bm*GDH) was kindly provided by Dr. Dirk Holtmann (Dechema, Germany) in the pETDuet vector (Novagen) cloned in the second multiple cloning site via *Nde*I and *Xho*I. The *Bm*GDH ORF was subsequently sub-cloned to pET-28b(+) to generate an N-terminally histidine tagged variant using the same restriction sites. The BVMO_AFL838__K511A mutant was prepared using site-directed mutagenesis with the QuickChange (Stratagene) method using primers K511A_F (5’ CAA CAT TCC GGG CGC GCC TGT TCA ATC ATT G 3’) and a complementary reverse primer. *E*. *coli* strains were routinely grown in LB medium containing ampicillin (0.1 mg.mL^-1^) or kanamycin (0.03 mg.mL^-1^) at 37°C with shaking (200 rpm).

### Protein Expression and Purification

*E*. *coli* BL21Gold(DE3) harbouring the pET-22:BVMO_AFL838_CTH or pET-28:*Bm*GDH plasmid was inoculated into ZYP5052 expression medium [20] containing 0.1 mg.mL^-1^ ampicillin or 0.03 mg.mL^-1^ kanamycin respectively. Cells were grown for 24–48 h at 20°C where after they were harvested through centrifugation (8 000 *xg*, 10 min) and resuspended in 50 mM Tris-HCl (pH 7.4) buffer containing 0.5 M NaCl and 20–30 mM imidazole (binding buffer). Cells were broken by a single passage through a continuous cell disrupter (Constant Systems) using 30 kPsi at 4°C and the crude cell-free extract was obtained through ultracentrifugation (100 000 x*g*, 90 min). The soluble fraction was loaded onto a 5 mL HisTrap FF Ni-affinity column (GE Healthcare) equilibrated in binding buffer. Unbound proteins were removed by washing with 10 column volumes of the same buffer. Proteins were eluted in the same buffer using a linear gradient of increasing imidazole concentration. Fractions containing the protein of interest were pooled and concentrated to approximately 2 mL through ultrafiltration using a 30 kDa NMWL Amicon Ultrafiltration unit. The concentrated BVMO_AFL838_CTH protein was soaked overnight in excess FAD. Concentrated protein samples were loaded onto a Sephacryl S100HR size exclusion column (GE Healthcare). Proteins were eluted in 10 mM Tris-HCl buffer (pH 8) containing 100 mM NaCl. Purified proteins were evaluated on SDS-PAGE using PageRuler Prestained Protein Ladder (ThermoScientific) and stained with Coomassie brilliant blue R-250. Protein concentrations were determined using the Bradford protein assay (Bio-Rad). FAD content was determined by incubation of the protein sample (typically 5 mg.mL^-1^) in ~ 8 M urea for 30 min, followed by spectrophotometric quantification using the extinction coefficient of 11.3 mM^-1^.cm^-1^ at 450 nm.

### Biotransformations and Steady-state kinetics

Biotransformations were performed in amber glass vials (40 mL) in a total reaction volume of 1 mL. Whole-cell (WC) and cell-free extract (CFE) biotransformations were performed as previously described [7]. Reactions with purified BVMO were performed in 100 mM Tris-HCl buffer (pH 8) containing 2 μM BVMO, 0.5 U *Bm*GDH, 100 mM glucose, 0.3 mM NADP^+^ and 10 mM substrate. Reactions were maintained at 20°C with shaking (200 rpm), where after they were extracted using an equal volume (2 x 0.5 mL) ethyl acetate containing either 2 mM 1-undecanol or 3-octanol as internal standard. GC-FID (and GC-MS for product identification) was performed on a Finnigan Trace GC ultra (ThermoScientific) equipped with a FactorFour VF-5ms column (60 m x 0.32 mm x 0.25 μm, Varian). Steady-state kinetics were performed by monitoring the oxidation of NADPH spectrophotometerically at 340 nm (ε_340_ = 6.22 mM^-1^.cm^-1^) or 370 nm (ε_370_ = 2.70 mM^-1^.cm^-1^). To investigate optimal pH, temperature, stability and effect of organic solvents, reactions typically contained 2 μM BVMO, 0.3 mM NADPH, 1 mM phenylacetone, 1% (v/v) methanol (100 mM Tris-HCl, pH 8; 25°C).

### Crystallization and Structure Determination

Crystals were grown using hanging-drop vapour-diffusion in 1 μL drops consisting of equal volumes of 6 mg.mL^-1^ BVMO and reservoir solution (0.1 M Tris-HCl pH 9, 1.8 M ammonium sulphate) and 5 x molar excess of FAD and NADP^+^ at 289 K. Yellow crystals grew within 2 weeks. Crystals were soaked in reservoir solution containing 30% glycerol prior to cryocooling. X-ray diffraction data (Table 1) were collected at Diamond (UK) on beamline I04-I. Data was processed using MOSFLM [21] and POINTLESS [22], with intensities scaled and merged using SCALA [22] from the CCP4 suite of programs. Molecular replacement was performed using Phaser [23] with PAMO (PDB:1W4X) as search model. Refinement was performed through iterative cycles of manual model building in COOT [24] and TLS and restrained refinement using Refmac [25]. Structures were validated using programs within the CCP4 suite [26]. Figures were generated in PyMOL.

**Table 1:** Data Collection and Refinement Statistics.

**Data Collection and Processing**	
Beamline	I04-I
Wavelength (Å)	0.9174
Resolution (Å)	32.4–1.9 (2.0–1.9)
Space group	C 2 2 2
Unit cell parameters	a = 109.67Å b = 177.51Å c = 76.37Å α = 90° β = 90° γ = 90°
Unique reflections	58733 (8465)
Completeness (%)	99.6 (99.6)
Mn(I)/sd(I)	15.3 (3.0)
Multiplicity	6.2 (6.3)
*R*_merge_	0.081 (0.669)
**Refinement**	
R_work_/R_free_^a^	0.1571/0.2046
Molecules in ASU	1
Average B-factor, all atoms (Å^2^)	28.0
r.m.s.d. Bond lengths (Å)	0.031
r.m.s.d. Bond angles (°)	2.71
Ramachandran distribution (%)^b^	96.9/2.7/0.4

Values for the highest resolution shell given in parenthesis

^a^ R_free_ calculated from 5% reflections omitted from structure refinement

^b^ Favoured/Permitted/Disallowed

### Accession numbers

Coordinates and structure factors for BVMO_AFL838_ have been deposited in the Protein Data Bank (PDB) under accession number 5J7X.

## Results and Discussion

### Purification and Characterization

**Fig 1:**
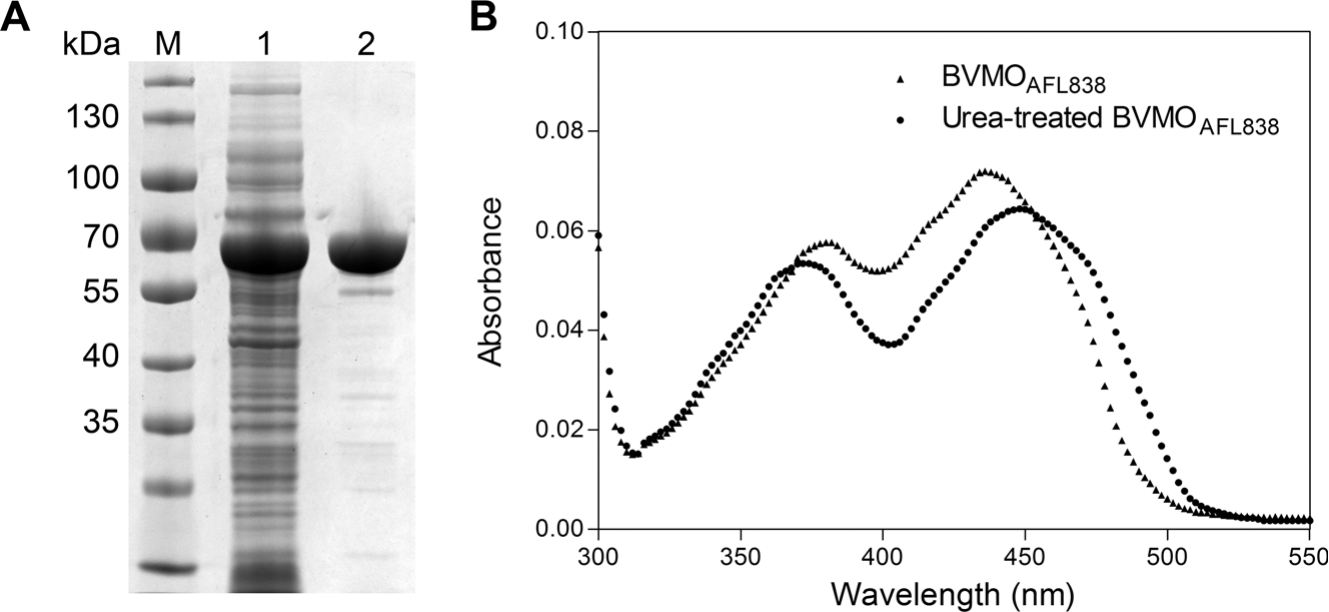
**(A) SDS-PAGE analysis of purified BVMO**_**AFL838**_. Lane M: molecular weight marker, Lane 1: *E*. *coli* cell-free extract expressing BVMO_AFL838_, Lane 2: Purified BVMO_AFL838_. **(B) Absorbance spectra of BVMO**_**AFL838**_ **before and after treatment with 8 M urea.** Extinction coefficient of free FAD at 450 nm: 11.3 mM^-1^.cm^-1^, Enzyme bound FAD at 436 nm: 12.7 mM^-1^.cm^-1^ and 11.6 mM^-1^.cm^-1^ at 450 nm.

**Fig 2:**
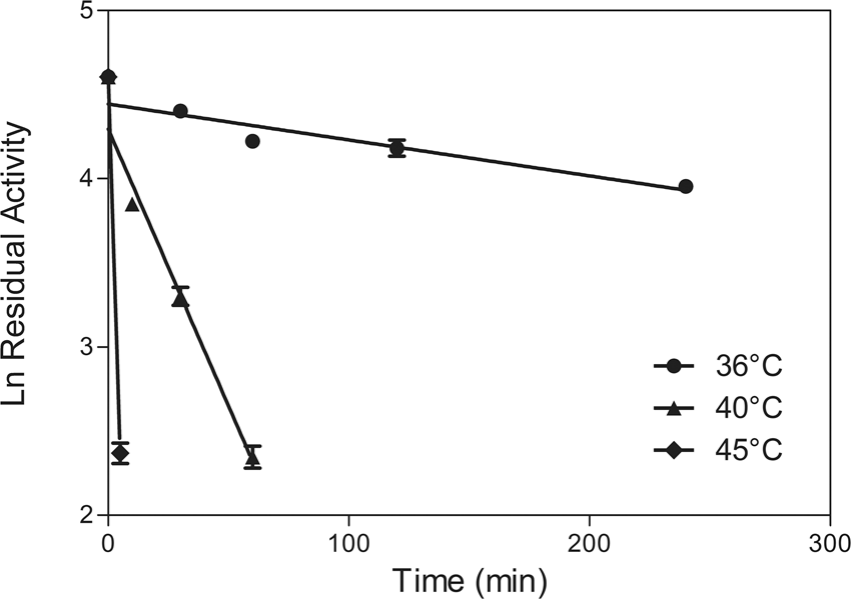
Temperature stability of BVMO_AFL838_.

BVMO_AFL838_ was heterologously expressed in *E*. *coli* as a C-terminally 6x histidine tagged protein and purified to near homogeneity using Ni-affinity and size exclusion chromatography (SEC). SDS-PAGE analysis confirmed the monomer molecular weight of 62.2 kDa (Fig 1A). SEC also showed BVMO_AFL838_ to exist as a monomer in solution. BVMO_AFL838_ was recovered with only 70–80% of bound flavin, but near full occupancy of the FAD could be achieved by soaking with excess FAD before SEC (Fig 1B). BVMO_AFL838_ was optimally active at pH 9, but retained approximately 80% activity at pH 8–8.5 and 60% at pH 7.5 and 9.5. Although the optimal temperature for BVMO_AFL838_ was found to be 40°C, the enzyme was rapidly inactivated at 40°C, with a half-life of only 21 min (Fig 2). Of the organic solvents tested, methanol showed to least affect activity, with nearly a 100% relative activity in up to 5% (v/v).

### Substrate specificity

**Fig 3:**
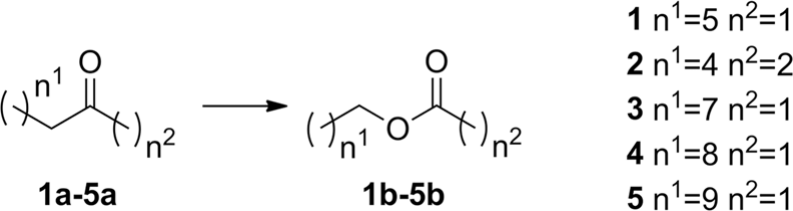
Baeyer-Villiger oxidation of aliphatic ketones.

**Fig 4:**
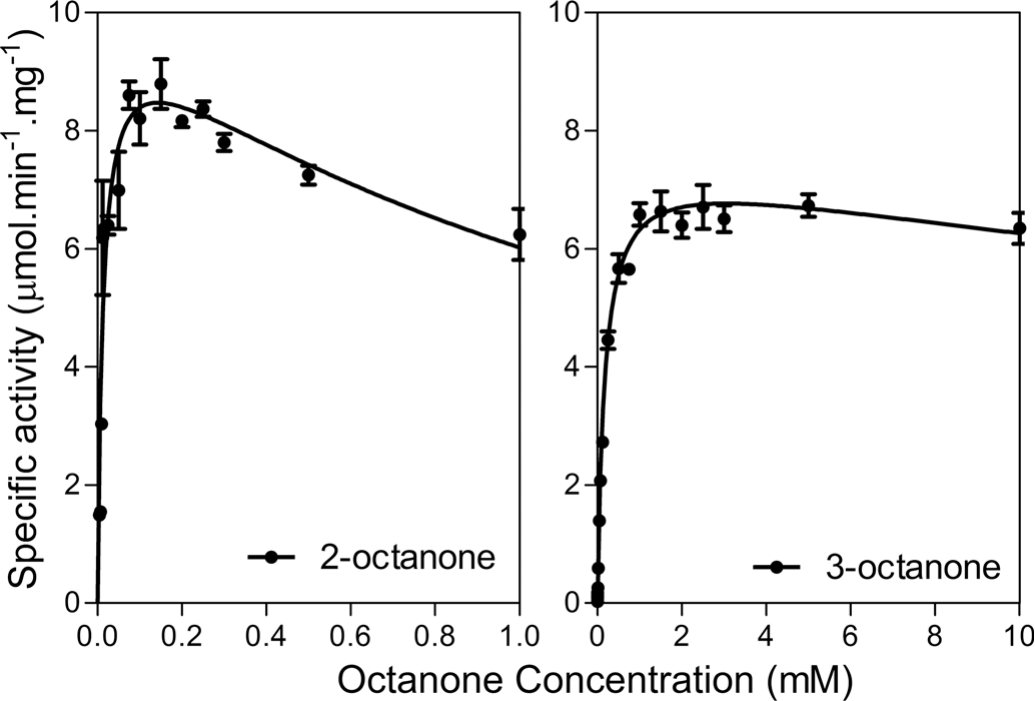
Steady-state kinetics of BVMO_AFL838_ with octanone.

We have previously shown BVMO_AFL838_ to be active towards aliphatic ketones and aryl ketones, with very few monocyclic ketones converted during whole-cell biotransformations [7]. Purified enzyme was tested using glucose dehydrogenase from *Bacillus megaterium* (*Bm*GDH) for cofactor regeneration to eliminate the possible uptake/transport limitations or toxicity of substrates or products toward *E*. *coli* whole cells. Similar to the whole-cell biotransformations which we previously reported, efficient conversion of the 2-alkanones was observed with nearly complete conversion of the C8-C12 substrates (10 mM of **1a, 3-5a**) within 8 h and even within 2 h for C8 (**1a**), with absolute regioselectivity forming the alkyl acetate products (Fig 3, Table 2). Turnover frequency (TOF) decreased with increasing chain-length but this could possibly be attributed to a decrease in solubility. Complete conversion of 3-octanone (**2a**) was also observed after 2 h, and observed TOF (*k*_obs_) values for 10 mM 2-octanone (**1a**) and 3-octanone were 5.3 s^-1^ and 6.6 s^-1^ respectively. To better investigate the effect of the position of the ketone group, steady-state kinetics were performed (Fig 4). A much lower *K*_M_ was observed for 2-octanone compared to 3-octanone, and although both substrates suffered from substrate inhibition, 2-octanone’s inhibition was more pronounced (*K*_i_ = 1.5 ± 0.4 mM) leading to lower TOF at high (10 mM) substrate concentrations (Table 2). Conversions of aliphatic ketones with substrate concentrations of up to 25 mM have been reported for BVMO3 from *Dietzia* sp. D5 [27], however no kinetic data was reported to evaluate reaction rates or possible substrate inhibition.

**Table 2:** Biotransformations of aliphatic ketones (10 mM) by purified BVMO_AFL838_ (2 μM) and kinetic parameters determined from initial velocities of NADPH oxidation.

Substrate		Conversion (%)		TOF (s^-1^)	*K*_m_ (mM)
		2h	8h	10 mM *k*_obs_	
**1a**	2-octanone	>99	>99	5.3	0.01 ± 0.003
**2a**	3-octanone	>99	>99	6.6	0.17 ± 0.03
**3a**	2-decanone	87	>99	6.8	<0.01
**4a**	2-undecanone	42	98	6.1	<0.01
**5a**	2-dodecanone	21	99	5.3	<0.01

Investigation into BVMO_AFL838_’s affinity for longer chain 2-alkanones (**3-5a**) revealed *K*_m_ values lower than 0.01 mM and less substrate inhibition than with 2-octanone with TOF reaching 6.8 s^-1^ (6.6 U.mg^-1^) for 2-decanone at 10 mM. Wild-type PAMO has been shown to accept aliphatic ketones [17] such as 2-dodecanone with a turnover frequency of 0.23 s^-1^ (*K*_m_ = 0.26 mM) and a quadruple mutant (15-F5) with improved activity towards 2-octanone (*k*_cat_ 2.3 s^-1^, *K*_m_ = 0.25 mM) has also been reported [28]. BVMOs from *Pseudomonas putida* KT2440 [29] and *P*. *veronii* MEK700 (MekA) [30] have also been shown to efficiently convert various aliphatic ketones, with specific activities for MekA between 0.45–0.89 U.mg^-1^ for C8-C12 2-alkanones.

**Fig 5:**
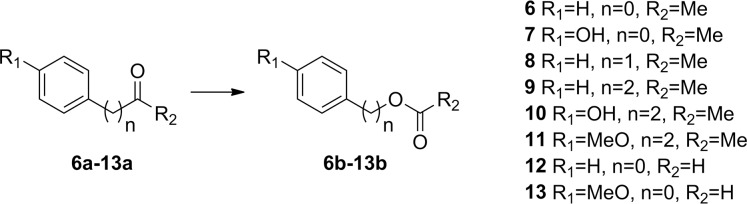
Baeyer-Villiger oxidation of aryl ketones and aldehydes.

**Fig 6:**
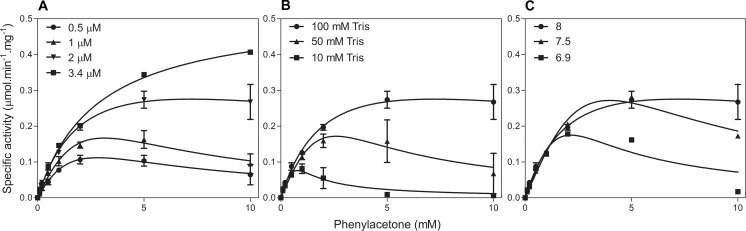
Steady-state kinetics of BVMO_AFL838_ with phenylacetone at different enzyme concentrations (A), Tris buffer concentrations (B) and pH values (C).

A good correlation was observed between whole-cell biotransformations and biotransformations using purified enzymes for aryl ketones, except no conversion was observed for phenylacetone (**8a**) and benzaldehyde (**12a**) using purified enzyme (Fig 5, Table 3). Very low TOF (0.3 s^-1^) were observed against 10 mM phenylacetone and steady-state kinetics revealed severe substrate inhibition with phenylacetone especially at lower enzyme concentrations (Fig 6A). Incubation of BVMO_AFL838_ with excess phenylacetone followed by reduction with NADPH did not affect the initial FAD reduction (NADPH binding), suggesting inhibition occurs during one of the intermediate steps of catalysis. Substrate inhibition by some acetophenone and benzaldehyde derivatives have also been observed with HAPMO [31] with a strong correlation between substrate affinity (*K*_m_) and degree of substrate inhibition. Substrate inhibition of BVMO_AFL838_ was also more pronounced at lower buffer concentrations and pH values (Fig 6B and 6C) to an extent that no observable rate could be detected. Glucose dehydrogenase (GDH) was used for cofactor regeneration in the biotransformations with purified enzyme and GDH is known to rapidly acidify the reaction mixture [32]. This type of fast acidification is not found with whole-cell biotransformation using glucose for cofactor regeneration through central metabolism. In addition, higher enzyme concentrations in whole-cells and potentially reduced effective substrate concentration due to diffusion/uptake limitations to the cytoplasm may also alleviate the substrate inhibition observed when using purified enzyme. The initial rates (measured as NADPH oxidation) were not maintained even at high substrate concentrations, suggesting also product inhibition.

**Table 3:** Biotransformations of aryl ketones and aldehydes by *E*. *coli* whole cells expressing BVMO_AFL838_ (WC) and purified BVMO_AFL838_ (ENZ). Kinetic parameters determined from initial velocities obtained from NADPH oxidation.

Substrate		Conversion (%)			TOF (s^-1^)	*K*_m_ (mM)
		WC_2h	ENZ_2h	ENZ_8h	10 mM *k*_obs_	
**6a**	Acetophenone	19	4	13	0.05	10.0 ± 1.2
**7a**	4-Hydroxyacetophenone	84	>99	>99	1.4	0.8 ± 0.05
**8a**	Phenylacetone	72	n.c.	n.c.	0.28^a^	2.4 ± 1.2^a^
**9a**	4-Phenyl-2-butanone	64	>99	>99	2.4	<0.01^b^
**10a**	4-(4-Hydroxyphenyl)-2-butanone	85	>99	>99	2.8	<0.01^b^
**11a**	4-(4-Methoxyphenyl)-2-butanone	68	85	85	4.3	<0.01^b^
**12a**	Benzaldehyde	62	n.c.	n.c.	0.03	6.5 ± 0.7
**13a**	4-Methoxybenzaldehyde	63	81	88	1.0	0.03 ± 0.002

^a^ Determined using 2 μM of BVMO

^b^ Substrate inhibition observed, *K*_i_>>*K*_m_

Similarly, no conversion was observed with benzaldehyde (**12a**) as substrate using purified enzyme although more than 60% conversion was observed using whole-cell biotransformations. Steady-state kinetics gave very low TOFs but no substrate inhibition was observed up to a concentration of 30 mM. Only slight substrate inhibition (*K*_i_ = 41 ± 6 mM) was observed with the *para*-substituted 4-methoxy benzaldehyde (**13a**) with more than 80% conversion observed after 2 h. Likewise, acetophenone with a hydroxyl substitution on the *para* position (**7a**) showed complete conversion after only 2 h, compared to 4% for acetophenone (**6a**). The substitutions on the phenyl ring lowered the *K*_m_ values with more than an order of magnitude, with TOF increased to more than 1 s^-1^ (Table 3). Increasing the distance between the ketone and phenyl group by a single carbon (**9**-**11a**), dramatically increased BVMO_AFL838_’s affinity for the substrates, with TOF now between 6 and 8 s^-1^ at 0.1 mM (2.4–4.3 s^-1^ at 10 mM) and again the *para*-substituted substrates (**10a** and **11a**) giving higher rates. Similar observations that substituents on the *para* position appear to be critical for substrate recognition have been made for 4-hydroxyacetophenone monooxygenases (HAPMOs) from both *Pseudomonas fluorescens* ACB [33,34] and *Pseudomonas putida* JD1 [35].

Absolute regioselectivity was also observed against all the aryl ketones tested. The ester products (**12b, 13b**) from benzaldehyde and 4-methoxybenzaldehyde were rapidly hydrolyzed to form phenol and 4-methoxyphenol (mequinol) as sole products. This regioselectivity is again similar as to what has been observed for HAPMO with various benzaldehyde derivatives [31], where even benzaldehydes with electron-withdrawing substituents on the aromatic ring prefers the formation of phenols.

### Overall structure of BVMOAFL838

The crystal structure of BVMO_AFL838_, the first of a fungal BVMO, was determined at a resolution of 1.9 Å. BVMO_AFL838_ shares more than 40% sequence identity with PAMO, which was used as search model for molecular replacement. Despite numerous attempts to co-crystallize BVMO_AFL838_ with co-factor and substrates, none of the crystals analyzed showed any electron density for bound NADP^+^ or the substrates used. A similar observation was made during the crystallization of PAMO [10], where the excess ammonium sulphate used as precipitant (crystallization agent) prevents the binding of NADP^+^. The first eight amino acids of the N-terminus were not modelled as it was not resolved in the electron density. Electron density was also not found for residues 230–239 which typically folds into a short α-helix on the surface of other BVMOs on the opposite side of the substrate entry (NADPH binding) channel.

**Fig 7:**
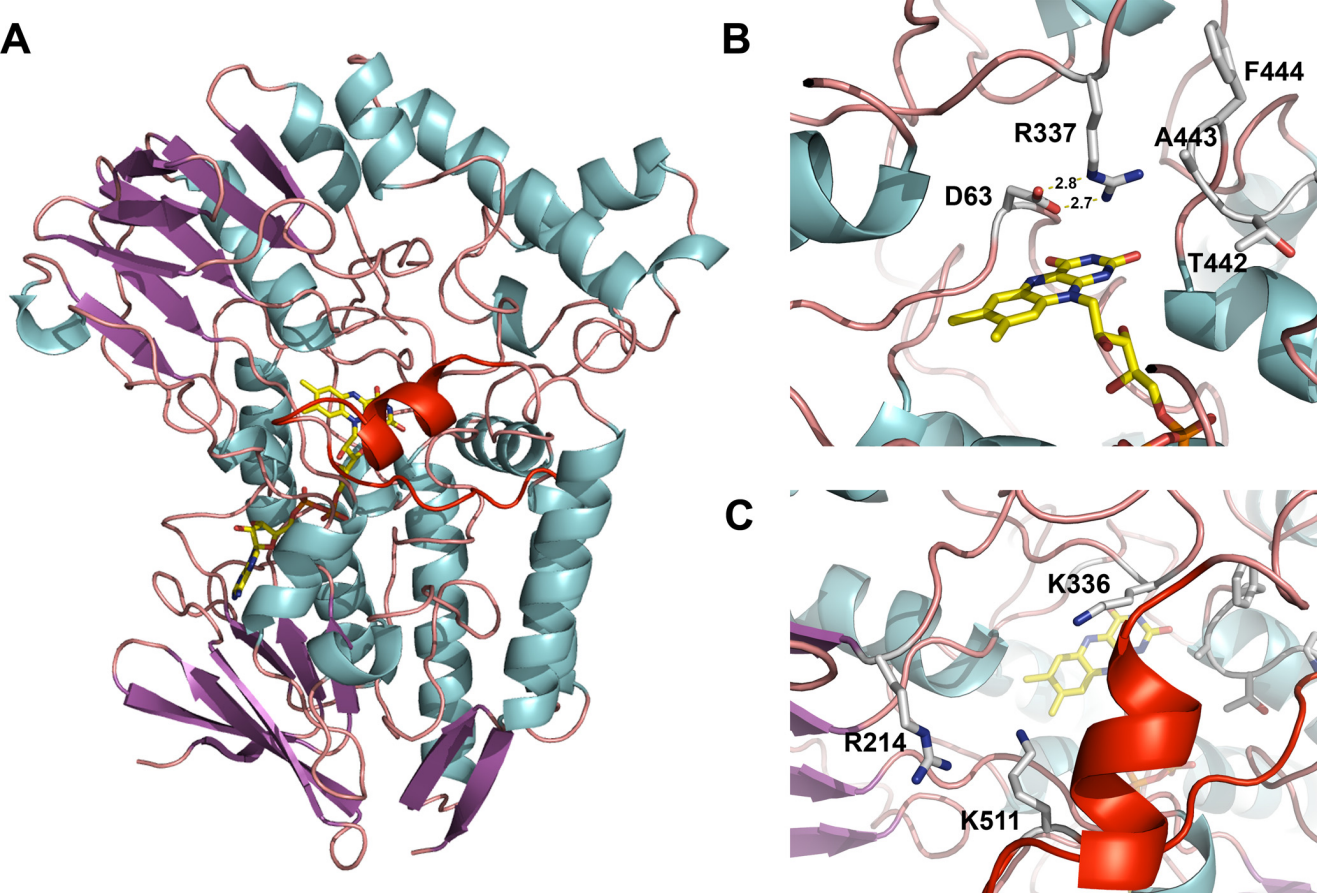
BVMO_AFL838_ crystal structure. (A) Overall structure of BVMO_AFL838_ represented as a ribbon diagram with bound FAD as a stick model. “Variable” loop region covering the active site entry channel is shown in red. (B) Active site of BVMO_AFL838_ showing the catalytically important Arg (R337) and Asp (D63) and bulge-structure (T442-F444). (C) Substrate entry channel showing the “variable” loop structure in red and residue K511 from the “variable” loop within close proximity to the anticipated 2’-phosphate of NADPH. Also shown are residues (R214, K336) previously implicated in co-factor (NADPH) recognition and binding.

BVMO_AFL838_, similar to the four bacterial BVMOs, displays two Rossmann fold domains, representing the FAD and NADP binding domains, with the isoalloxazine ring of FAD positioned at the domain interface (Fig 7A). The FAD is bound with the *re*-face exposed to the substrate binding pocket and the *si*-face on top of a conserved Tyr69 and Trp52 forming conserved hydrogen bond interactions with the ribitol moiety of FAD. Except for the conserved Rossmann fold signature motif (GXGXXG) the region binding the rest of the FAD molecule is less conserved as most of the contacts are with main chain amide groups and via water mediated interactions. Despite this, Val115 is absolutely conserved between the five known BVMO structures, with two hydrogen bonds forming between the adenine of FAD and the main chain amide and carbonyl group of Val. The previously identified BVMO-signature motif [36] is absolutely conserved in position and sequence.

### Active site architecture

The catalytic site Arg337 and Asp63, implicated in stabilization of the peroxyflavin intermediate and positioning and guiding the cofactor and substrate during catalysis, are conserved. The Arg is located in the “in” position and forms two salt bridges with Asp (Fig 7B). Similar to PAMO, a bulge (residues 442–444) protrudes into the active site of BVMO_AFL838_ (Fig 7B). Mutation studies showed that elimination of this bulge increased the substrate scope of PAMO [37]. However, STMO and OTEMO display similar bulges, but accept progesterone and small monocyclic ketones as substrates respectively.

### Variable loop structure

Partially covering the substrate entry channel of BVMO_AFL838_ is a loop region (residues 449 to 514) of which the first half forms an α-helix structure (Fig 7C). This mobile loop region is typically disordered (CHMO, STMO) or forms an unstructured loop (PAMO) or a β-hairpin structure (OTEMO) that is solvent exposed and positioned away from the active site channel in the “resting” state. In BVMO_AFL838_ the mobile/“variable” loop adopts a position similar to that in the CHMO “closed” structure, where part of the active site entry channel is closed off. In this conformation however, the conserved Trp502 (W492 CHMO numbering) is positioned away from the active site and the NADPH cofactor, suggesting a rearrangement of this loop upon cofactor and substrate binding. Apart from Arg214 and Lys336 that have been implicated in NADPH binding, the conformation of the mobile loop also positioned Lys511 within close proximity to the anticipated 2’-phosphate of NADPH as suggested through superimposition with other BVMOs. To probe whether the conformation of the variable/mobile loop observed in the structure is physiologically important, K511 was mutated to an Ala. Although still catalytically active, the K511A mutant showed reduced catalytic activity towards 3-octanone as well as an increased uncoupling of NADPH oxidation and product formation.

## Conclusions

BVMO_AFL838_ has a clear preference towards aliphatic ketones and phenyl substituted 2-alkanones (aryl ketones where the alkyl chain is at least two carbons between the phenyl and carbonyl group). To our knowledge, this data represents the highest specific rates obtained to date for BVMO transformations of 2-alkanones. Moreover, biotransformations of linear and aryl ketones using purified enzyme compared to whole-cell biotransformations highlights the importance of kinetic studies as opposed to single-concentration conversions to determine the substrate scope and preference of BVMOs when working with purified enzymes as these systems are more prone to substrate inhibition. BVMO_AFL838_ constitutes the first structural investigation of a fungal BVMO. The crystal structure of BVMO_AFL838_ revealed an overall fold similar to bacterial BVMOs. More extensive crystallization trials is currently underway to find conditions to allow solving BVMO_AFL838_ with NADP^+^ and substrates to further investigate conformations of the “variable” loop region, as well as determinants of substrate scope and regioselectivity. More data is currently needed to understand the discrepancy between substrate pocket plasticity to accommodate a wide range of substrates yet high product selectivity.
